# Higher levels of physical activity are independently associated with a lower incidence of diabetic retinopathy in Japanese patients with type 2 diabetes: A prospective cohort study, Diabetes Distress and Care Registry at Tenri (DDCRT15)

**DOI:** 10.1371/journal.pone.0172890

**Published:** 2017-03-03

**Authors:** Hirohito Kuwata, Shintaro Okamura, Yasuaki Hayashino, Satoru Tsujii, Hitoshi Ishii, for the Diabetes Distress and Care Registry at Tenri Study Group

**Affiliations:** 1 Department of Diabetology, Nara medical University, Kashihara, Nara, Japan; 2 Department of Endocrinology, Tenriyorozu Hospital, Tenri, Nara, Japan; German Diabetes Center, Leibniz Center for Diabetes Research at Heinrich Heine University Düsseldorf, GERMANY

## Abstract

We assessed the prospective association between baseline levels of physical activity (PA) and the incidence of newly developed diabetic retinopathy (DR) in patients with type 2 diabetes. Data from 1,814 patients with type 2 diabetes without DR were obtained from a Japanese diabetes registry at Tenri Hospital, Nara, Japan. To assess the independent correlations between baseline PA levels and newly developed DR, the participants were divided into five categories based on their PA levels. A Cox proportional hazards model with time-varying exposure information was used and adjusted for potential confounders to assess the independent correlations. At baseline, the mean age, BMI, and hemoglobin A1c levels of the patients were 65.5 years, 24.5 kg/m^2^, and 7.2% (54 mmol/mol), respectively. After 2 years, newly developed DR was confirmed in 184 patients (10.1%). Patients with newly developed DR had longer duration of type 2 diabetes (14.7 versus 11.0 years, p < 0.0001), higher systolic blood pressure (139.2 versus 135.1 mmHg, p = 0.0012), lower estimated glomerular filtration rate (74.0 versus 77.1 mL/min/1.73 m^2^, p = 0.0382), greater urinary albumin–creatinine ratio (4.00 versus 2.45 mg/mmol, p < 0.0039), and higher HbA1c levels (7.5 versus 7.2%, p = 0.0006) than those without newly developed DR. The multivariable-adjusted hazard ratios for DR development were 0.87 (95% CI, 0.53–1.40; p = 0.557), 0.83 (95% CI, 0.52–1.31; p = 0.421), 0.58 (95% CI, 0.35–0.94; p = 0.027), and 0.63 (95% CI, 0.42–0.94; p = 0.025)for the second, third, fourth, and fifth PA categories, respectively, compared with the reference category of patients with a mean PA of 0 metabolic equivalent of task-hours/week). Higher PA levels are independently associated with a lower incidence of DR in Japanese patients with type 2 diabetes.

## Introduction

The prevalence of type 2 diabetes mellitus has significantly increased worldwide, which has in turn increased the burdens on individuals and health-care systems [1]. One such burden is the increased prevalence of chronic complications, such as diabetic retinopathy (DR). DR has an insidious onset and is a major cause of vision loss in patients aged 20–64 years [2]. Thus, effective diagnostic methods and therapeutic tools are needed to prevent DR in patients with diabetes. Identification of clinical features in patients with diabetes that can predict the development and progression of DR is crucial; suggested risk factors include a long history of diabetes, elevated blood glucose levels, increased blood pressure, and impaired kidney function [3–7].

Studies have shown that increased physical activity (PA) is associated with a substantially reduced risk of cardiovascular events [8,9] and that a low PA level is an independent predictor of all-cause mortality in patients with type 2 diabetes [10,11]. However, whether physical inactivity is a risk factor for DR or higher PA is associated with a reduced risk of developing DR remains unclear. Chronic running exercise was found to alleviate the early progression of nephropathy in rats [12]. In humans, studies in patients with type 1 diabetes have shown no significant association between PA and DR [13–15]. Conversely, the Wisconsin Epidemiologic Study of Diabetic Retinopathy (WESDR) showed that only female patients who engaged in team sports had a reduced risk of having proliferative DR [16]. Moreover, the Finnish Diabetic Nephropathy (FinnDiane) Study demonstrated a significant association between low PA levels and proliferative DR in patients with type 1 diabetes [17]. However, few large-scale prospective studies have evaluated the association between PA and newly developed DR in patients with type 2 diabetes.

We studied data from a large-scale single-center registry of Japanese patients with type 2 diabetes to elucidate the prospective relationship between baseline PA levels and the development of DR over a 2-year follow-up period.

## Materials and methods

### Patients

Patient data were derived from the second-year survey of the Diabetes Distress and Care Registry at Tenri [18–26], a study conducted at the Tenri Hospital, a regional tertiary-care teaching hospital in Nara, Japan. In brief, this cohort study was aimed at evaluating the cross-sectional and prospective associations between the psycho-socioeconomic factors behind, biomarkers of, therapeutic modalities for, and incidences of chronic complications in patients with diabetes. The registry recruited patients with diabetes who visited the outpatient clinic at the Tenri Hospital between October 2009 and December 2010. We conducted our survey from January to December in 2011, 2012, and 2013. We excluded patients diagnosed with prediabetes (by an oral glucose tolerance test), gestational diabetes, type 1 diabetes, or diabetes induced by steroid use or other endocrine diseases. These exclusions enabled us to obtain data from patients with type 2 diabetes. At registration, the attending physician confirmed the diagnosis according to the *Classification and Diagnostic Criteria of Diabetes Mellitus* by the Japan Diabetes Society. For this analysis, we included only patients with available baseline PA data who did not have DR. The ethics committee of the Tenri Hospital approved this study, and written informed consent was obtained from every participant before its initiation. Patient records were de-identified and analyzed anonymously.

#### Inclusion and exclusion criteria

**Fig 1:**
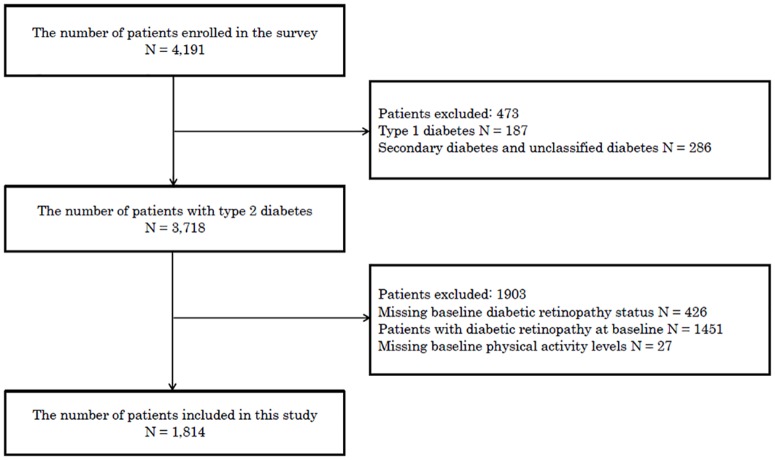
Inclusion and exclusion criteria of patients in this study. Fig 1 shows the inclusion and exclusion criteria for patients in this study.

For the analysis, we included only patients whose baseline PA data were available and who did not have DR in 2011. We began collecting data on DR in 2011; therefore, we used the 2011 data as the baseline data in this study. In 2011, of the 4,330 eligible patients with diabetes (mean age, 65.6 ± 12.1 years; 40.5% were female; 4.6% had type 1 diabetes; and 92.3% had type 2 diabetes), 4,191 provided consent to participate in this study. In total, 3,718 were confirmed to have type 2 diabetes. Twenty-seven patients were excluded because of missing baseline PA data, and 426 patients were excluded because of missing baseline DR status data. Furthermore, we excluded 1,451 patients who already had DR at baseline. The remaining 1,814 patients were included in this study (Fig 1).

### Data collection

In the survey, patients underwent routine inquiries about their medical history, a physical examination, and laboratory tests. The following demographic data were collected: age, sex, height, body weight, systolic blood pressure (sBP), diastolic blood pressure (dBP), smoking status, and alcohol consumption. Medical history, including micro- and macrovascular complications, and treatment modalities, including medication for hypertension, were also recorded. Laboratory tests included the evaluation of hemoglobin A1c (HbA1c) levels, lipid profiles, serum creatinine and uric acid levels, high-sensitivity C-reactive protein (hs-CRP) levels, and the urinary Albumin:creatinine ratio from a spot urine sample. HbA1c levels were expressed in accordance with the National Glycohemoglobin Standardization Program (NGSP) as recommended by the Japanese Diabetes Society [27] and in NGSP and International Federation of Clinical Chemistry units. The estimated glomerular filtration rate (eGFR) was calculated using the following equation proposed by the Japanese Society of Nephrology [28]:

$$GFR\left(mL/\text{min}\cdot 1.73m^{2}\right)=194\times Cr^{-1.094}\times age^{-0.287}\left(\times 0.739\begin{array}{ccc} for & female & patients \end{array}\right)$$

### Outcomes

DR status was evaluated at 1, 2, and 3 years after registration. The relationship between PA and DR development was the primary concern. We requested all the patients to receive an ophthalmological consultation every year. DR was assessed using a dilated fundus examination conducted by ophthalmologists blinded to the systemic parameters of this study. During the follow-up period, patients who did not receive a fundus examination were not excluded. Patients with good glycemic control are less likely to consult ophthalmologists; therefore, we believed that overestimation of the incidence of DR may result from the exclusion of these patients. In this study, DR grading was based on published literature and performed using the Fukuda scale [29]. We assessed whether DR had developed but did not evaluate detailed DR stages or whether it was non-proliferative or proliferative. The change in DR status and time point at which it occurred were recorded based on the results of a fundus examination by the end of 2013. In patients with an asymmetric DR manifestation, the most severe DR was selected for grading.

### Physical activity

PA was measured at baseline using a Japanese short version of the International Physical Activity Questionnaire (IPAQ) [30]. IPAQ assessed the duration and frequency of walking, moderate PA (MPA), and vigorous PA (VPA) lasting for at least 10 min in a typical week. We estimated PA by multiplying the reported duration (h) per week of walking, MPA, and VPA by their respective metabolic equivalent of tasks (METs; walking = 3.3 METs; MPA = 4 METs; and VPA = 8 METs) to obtain the estimated energy expenditure in MET-hours/week [31]. Using these values, total moderate-to-vigorous PA (MVPA) was defined as follows [32]:

$$7days\times \left(3.3METs\times \begin{array}{cc} walking & hours/day+4METs\times \begin{array}{cc} MPA & hours/day+8METs\times \begin{array}{cc} VPA & hours/day \end{array} \end{array} \end{array}\right)$$

### Statistical analysis

Continuous variables were expressed as means and standard deviation or medians and interquartile ranges (IQR) for variables with non-normal distribution. Intergroup differences between five PA categories were evaluated using one-way ANOVA or the Wilcoxon rank-sum test. An unpaired two sample Student’s *t*-test was used to compare continuous variables between patients with and without newly developed DR. Fisher’s exact test or the chi-squared test was used to examine categorical variables.

Total MVPA was categorically evaluated. To define the categories, we chose an MVPA cutoff of 8.25 MET-hours/week, corresponding to the American Diabetes Association recommendation of 150 min/week of MVPA (brisk walking in this case) [33]. For those with ≥8.25 MET-hours/week, we used tertiles within this sufficiently active group to determine further cutoffs (23.1, 55.1). Finally, the participants were divided into five categories: mean PA levels (in MET-hours/week) of 0, 4.8 (IQR, 3.3–6.6), 13.2 (IQR, 11–16.5), 26.4 (IQR, 23.1–34.6), and 77 (IQR, 55.1–128.3).

The associations between PA categories and DR development were analyzed using the Cox proportional hazards model with time-varying exposure information, considering clustering within attending physicians. The relationship between baseline PA and DR development was estimated in a cohort of patients without DR at baseline. The person-time was calculated as the period between the registration date until the day the outcome was confirmed or the end of follow-up (whichever occurred first). The hazard ratio (HR; 95% CIs) was estimated for the outcome in comparison with a reference category of patients with a mean PA of 0 MET-hour/week. In the analysis, the following three statistical models were used: a crude model; an age- and sex-adjusted model; and a model adjusted for age, sex, BMI, sBP, dBP, heart rate, high-density lipoprotein (HDL) levels, low-density lipoprotein levels, triglyceride levels, eGFR, HbA1c levels, duration of diabetes, diabetes therapy, and history of cardiovascular disease (CVD). We selected these covariates because they are known to be associated with PA and DR. In this multivariable-adjusted Cox proportional hazards model, sBP, dBP, BMI, and HbA1c were evaluated as time-varying exposure information. We adjusted sBP, dBP, BMI, and HbA1c levels as time-varying variables using data from the second and third year observation periods of this study. We selected these covariates as time-varying variables because they are known to vary over time and are associated with DR. Conversely, we did not evaluate PA as a time-varying variable because PA was not evaluated every year in this investigation. All *p* values were two-sided, and *p* values <0.05 were considered to be statistically significant. All analyses were performed using Stata/SE version 12.1 (StataCorp LP, College Station, TX, USA).

## Results

### Patient characteristics

Table 1 displays the demographic characteristics and laboratory data of the patients according to their PA categories. At baseline, their mean age was 65.5 years, mean BMI was 24.5 kg/m^2^, and mean HbA1c level was 7.2% (54 mmol/mol). Patients with higher PA tended to be younger (*p* = 0.0001), predominantly male (*p* < 0.0001), and have a lower BMI (*p* = 0.0118), lower resting heart rate (*p* = 0.0003), higher HDL levels (*p* = 0.0056), and lower hs-CRP levels (*p* = 0.0415). A lower percentage of them had never smoked, a higher percentage of them had a history of smoking (*p* < 0.0001), and a lower percentage of them had a history of CVD (*p* < 0.0001).

**Table 1:** Baseline participant characteristics for each of the physical activity categories^a^.

		Physical activity level categories					
	All subjects	Category 1	Category 2	Category 3	Category 4	Category 5	
	*n* = 1814	*n* = 245	*n* = 340	*n* = 431	*n* = 388	*n* = 410	*p* value
Physical activity levels, MET-hours/week							
Median (interquartile range)	16.5 (5.5–37.1)	0	4.8 (3.3–6.6)	13.2 (11–16.5)	26.4 (23.1–34.6)	77 (55.1–128.3)	
Range, MET-hours/week		0	0.3–8.24	8.25–19.82	19.83–41.99	42–	
Age, years	65.5 (11.5)	68.4 (11.7)	65.2 (13.2)	64 (11.5)	65.4 (10.9)	65.7 (10.2)	0.0001
Female, %	37.2	46.9	42.9	40.8	30.4	29.3	< 0.0001
Duration of diabetes mellitus, years	11.4 (8.6)	11.8 (9.5)	11.7 (9.1)	11.4 (8.1)	11.1 (8.2)	11.2 (8.3)	0.763
BMI, kg/m^2^	24.5 (4.3)	24.5 (4.3)	25.1 (4.5)	24.4 (4.3)	24.3 (4.3)	24 (3.9)	0.0118
Systolic blood pressure, mm Hg	135.6 (17.5)	134.1 (18)	135.4 (17.9)	136.2 (16.7)	136.2 (16.8)	135.3 (18.5)	0.5982
Diastolic blood pressure, mm Hg	73.6 (11.9)	72.4 (11.8)	73.5 (12)	74.3 (11.7)	74.7 (11.7)	72.8 (12.1)	0.0508
Heart rate, beats/min	73.6 (12.5)	74.2 (12.4)	75.3 (13.1)	73.9 (12.1)	73.8 (13)	71.3 (12)	0.0003
HDL, mmol/L	1.44 (0.4)	1.38 (0.39)	1.41 (0.38)	1.44 (0.4)	1.49 (0.42)	1.46 (0.38)	0.0056
LDL, mmol/L	2.69 (0.73)	2.69 (0.69)	2.74 (0.72)	2.7 (0.71)	2.67 (0.77)	2.65 (0.73)	0.4827
^b^Triglyceride, mmol/L	1.52 (1.04–2.12)	1.60 (1.09–2.11)	1.56 (1.09–1.56)	1.51 (0.06–2.13)	1.48 (1.03–2.12)	1.45 (0.98–2.14)	0.8481
Creatinine, μmol/L	69.6 (40.1)	69.5 (31.4)	73.8 (66.3)	67.5 (39.5)	69.6 (27)	68.5 (23.3)	0.2631
eGFR, mL/min/1.73m^2^	76.7 (22.2)	74.6 (24.4)	75.8 (24.3)	78.3 (21)	76.2 (20.8)	77.6 (21.2)	0.1952
^b^Urinary Albumin:creatinine ratio, mg/mmolCr	2.57 (1.42–6.52)	3.85 (1.83–9.27)	2.43 (1.30–6.78)	2.37 (1.33–5.67)	2.36 (1.39–5.53)	2.55 (1.47–6.79)	0.5121
ALT, IU/L	25.7 (19.7)	26 (18)	26.8 (22.3)	25.5 (19.1)	25.4 (18)	25.1 (20.4)	0.8052
AST, IU/L	26.9 (14.8)	27.5 (14.7)	26.8 (15.6)	26.6 (14.3)	26.6 (12.8)	27.4 (16.5)	0.8872
Gamma GTP, IU/L	45.7 (67.9)	45.8 (54)	43.5 (45.5)	44.6 (83.5)	50.1 (84.3)	44.4 (54)	0.6699
^b^Highly sensitive C-reactive protein, μg/L	800 (100–1600)	900 (500–2000)	900 (100–2000)	600 (100–1500)	700 (100–1500)	700 (100–1400)	0.0415
HbA1c							
NGSP, %	7.2 (1.1)	7.2 (1.1)	7.4 (1.1)	7.3 (1.1)	7.2 (1)	7.2 (1)	0.0204
IFCC, mmol/mol	54 (11)	53.3 (11.2)	55.7 (11.6)	54.1 (11.4)	53.1 (10.3)	53.6 (10.5)	0.0204
Smoking, %							< 0.0001
Never	41	50.2	44	43	36.3	35.1	
Past	41.8	29.4	38.1	40.7	48.7	47.1	
Current	17.2	20.4	18	16.3	15	17.8	
ACE inhibitor use, %	5	3.7	7.4	4.4	3.9	5.6	0.162
ARB use, %	31.6	34.3	31.2	30.2	30.4	33.2	0.740
Diabetes therapy, %							0.071
Diet only	19.4	17.1	15.7	19.0	22.3	21.2	
Oral medication only	54.5	53.5	52.5	55.0	55.1	55.9	
Insulin	26.1	29.4	31.8	26.0	22.6	22.9	
History of cardiovascular disease, %	20.4	30.2	18.2	16	23.7	17.8	< 0.0001

^a^Data are means ± standard deviation unless otherwise indicated

^b^Median and interquartile range

The differences in characteristics between patients with and without newly developed DR are presented in Table 2. We found significant differences between the two groups in terms of age (p = 0.006), duration of diabetes (*p <* 0.0001), sBP (*p =* 0.0012), resting heart rate (*p =* 0.0408), eGFR (*p =* 0.0382), UACR (*p =* 0.0039), and HbA1c levels (*p =* 0.0006). No significant differences were evident between patients with and without newly developed DR in sex, BMI, PA levels, dBP, lipid profiles, serum creatinine levels, smoking status, medication use including ACE inhibitors, ARB, and past history of CVD.

**Table 2:** Differences between patients with and without newly developed diabetic retinopathy over 2 years of follow-up^a^.

	Patients without development of diabetic retinopathy	Patients with development of diabetic retinopathy	*p*-value
Variables	*n* = 1630	*n* = 184	
Age, years	65.3 (11.6)	67.5 (10.6)	0.0060
Female, %	37.2	37.5	0.936
Duration of diabetes mellitus	11.0 (8.4)	14.7 (9.1)	<0.0001
BMI, kg/m^2^	24.5 (4.3)	24.3 (4.0)	0.3620
Physical activity levels, MET- hours/week ^b^	16.5 (5.5–38.5)	11.6 (4.1–32.9)	0.0581
Systolic blood pressure, mm Hg	135.1 (17.5)	139.2 (17.3)	0.0012
Diastolic blood pressure, mm Hg	73.7 (12.0)	72.7 (11.2)	0.1359
Heart rate, /min	73.4 (12.6)	75.1 (11.8)	0.0408
HDL, mmol/L	1.44 (0.40)	1.41 (0.40)	0.1501
LDL, mmol/L	2.69 (0.73)	2.66 (0.65)	0.2906
Triglyceride, mmol/L ^b^	1.52 (1.04–2.12)	1.46 (1.02–2.16)	0.4872
Creatinine, μmol/L	69.5 (41.4)	70.9 (26.3)	0.3229
eGFR, mL/min/1.73 m^2^	77.1 (22.1)	74.0 (22.8)	0.0382
Urinary Albumin:creatinine ratio, mg/mmol ^b^	2.45 (1.39–6.08)	4.00 (1.76–13.48)	0.0039
ALT, IU/L	25.9 (20.1)	24.0 (14.9)	0.1058
AST, IU/L	27.1 (15.1)	25.5 (11.6)	0.0885
Gamma GTP, IU/L	46.5 (69.9)	38.8 (46.2)	0.0744
Highly sensitive C-reactive protein, μg/L^b^	700 (100–1600)	800 (100–2100)	0.2787
HbA1c			
NGSP, %	7.2 (1.1)	7.5 (1.1)	0.0006
IFCC, mmol/mol	53.7 (10.9)	56.5 (11.7)	0.0006
Smoking, %			0.735
Never	41.2	39.1	
Past	41.5	44.6	
Current	17.3	16.3	
ACE inhibitor use, %	4.9	6.5	0.370
ARB use, %	30.9	38.0	0.054
History of cardiovascular disease, %	20.4	20.7	0.923

^a^ Data are means ± (standard deviation) unless otherwise indicated

^b^ Median and interquartile range

In the cohort of patients who were followed up for a median of 700 days, 184 developed DR [incidence ratio, 54.3/1000 person-years (95% CI, 47.0–62.7); Table 3]. In almost all these patients, their DR was non-proliferative. HRs calculated using the Cox proportional hazards model for the association between PA categories and DR development are shown in Table 4. We observed a significant association between baseline PA and subsequent DR development in all three models. HRs for developing DR in the second, third, fourth, and fifth PA categories compared with the first PA category were 0.84 (95% CI, 0.57–1.24; p = 0.373), 0.75 (95% CI, 0.55–1.04; p = 0.081), 0.49 (95% CI, 0.30–0.80; p = 0.004), and 0.57 (95% CI, 0.41–0.79; p = 0.001), respectively, for the crude model; 0.92 (95% CI, 0.59–1.43; p = 0.697), 0.84 (95% CI, 0.58–1.22; p = 0.367), 0.53 (95% CI, 0.33–0.85; p = 0.009), and 0.61 (95% CI, 0.44–0.85; p = 0.004), respectively, for the age- and sex-adjusted model; 0.89 (95% CI, 0.54–1.44; p = 0.628), 0.85 (95% CI, 0.55–1.32; p = 0.464), 0.58 (95% CI, 0.35–0.94; p = 0.026), and 0.67 (95% CI, 0.48–0.93; p = 0.019), respectively, for the multivariable-adjusted model; and 0.87 (95% CI, 0.53–1.40; p = 0.557), 0.83 (95% CI, 0.52–1.31; p = 0.421), 0.58 (95% CI, 0.35–0.94; p = 0.027), and 0.63 (95% CI, 0.42–0.94; p = 0.025), respectively, for the multivariable-adjusted model with time-varying exposure information. During the follow-up period, 149 patients who did not undergo a fundus examination were not excluded from this study; however, the numbers of these patients in the first, second, third, fourth, and fifth PA categories were 33, 33, 28, 30, and 25, respectively, and the same associations were evident when we excluded these patients. For example, the HRs for developing DR in the second, third, fourth, and fifth PA categories compared with the first PA category were 0.83 (95% CI, 0.51–1.35; p = 0.463), 0.83 (95% CI, 0.51–1.35; p = 0.461), 0.53 (95% CI, 0.33–0.85; p = 0.008), and 0.60 (95% CI, 0.40–0.90; p = 0.013), respectively, for the multivariable-adjusted model with time-varying exposure information. We thought that excluding these patients may overestimate the incidence of DR, so we did not exclude them; patients with good glycemic control are less likely to see ophthalmologists.

**Table 3:** Baseline physical activity categories and incidence of diabetic retinopathy.

Physical activity categories	Number of participants	Person-years (days)	Number of outcomes	Incidence ratio (95% CI) ^a^
Category 1	245	451.1	32	70.9 (50.2–100.3)
Category 2	340	629.4	43	68.3 (50.7–92.1)
Category 3	431	803.3	48	59.8 (45–79.3)
Category 4	388	735.3	29	39.4 (27.4–56.8)
Category 5	410	770.9	32	41.5 (29.4–58.7)

^a^Incidence of outcomes per 1000 person-years

**Table 4:** Association between physical activity levels and development of diabetic retinopathy.

	Physical activity categories				
	Category 1	Category 2	Category 3	Category 4	Category 5
Number of participants	*n* = 245	*n* = 340	*n* = 431	*n* = 388	*n* = 410
Physical activity levels, MET-hours/week					
Median (interquartile range)	0	4.8 (3.3–6.6)	13.2 (11–16.5)	26.4 (23.1–34.6)	77 (55.1–128.3)
Range, MET-hours/week	0	0.3–8.24	8.25–19.82	19.83–41.99	42–
Hazard ratio for development (95% CI)					
Crude model	Ref.	0.84 (0.57–1.24)	0.75 (0.55–1.04)	0.49 (0.30–0.80)	0.57 (0.41–0.79)
Age- and sex-adjusted model	Ref.	0.92 (0.59–1.43)	0.84 (0.58–1.22)	0.53 (0.33–0.85)	0.61 (0.44–0.85)
^a^Multivariate-adjusted model	Ref.	0.89 (0.54–1.44)	0.85 (0.55–1.32)	0.58 (0.35–0.94)	0.67 (0.48–0.93)
^b^Multivariate-adjusted model	Ref.	0.87 (0.53–1.40)	0.83 (0.52–1.31)	0.58 (0.35–0.94)	0.63 (0.42–0.94)

^a^Adjusted for age, sex, BMI, duration of diabetes mellitus, systolic blood pressure (sBP), diastolic blood pressure (dBP), heart rate (HR), Hemoglobin A1c (HbA1c) levels, high-density lipoprotein (HDL), low-density lipoprotein (LDL), triglyceride, estimated glomerular filtration rate (eGFR), diabetes therapy, and history of cardiovascular disease (CVD).

^b^Adjusted for age, sex, BMI, duration of diabetes mellitus, sBP, dBP, HR, HbA1c levels, HDL, LDL, triglyceride, eGFR, diabetes therapy, and history of CVD. SBP, dBP, BMI, and HbA1c were assumed as time-varying exposure information.

## Discussion

In this study, higher PA was independently associated with a lower incidence of DR in patients with type 2 diabetes. To the best of our knowledge, this is the first large-scale prospective study to evaluate the influence of PA on DR development in patients with type 2 diabetes.

The first large-scale study to examine the association between PA and DR was the Pittsburgh Insulin-Dependent Diabetes Mellitus Morbidity and Mortality Study, which was published in 1986 [13]. Previous studies have likewise shown no significant association between PA and DR in patients with type 1 diabetes [13–15]. However, the data indicated that exercise had no adverse effect on the progression and development of DR. In contrast, WESDR reported protective associations between PA and proliferative DR. However, this association was shown in only female patients who were diagnosed with diabetes before the age of 14 years [16]. Moreover, the FinnDiane study showed a significant association between low PA and a higher incidence of proliferative DR in patients with type 1 diabetes [17]. Although the FinnDiane study was conducted at a larger scale than the previous studies on the association of PA and DR, the generalizability of its findings was limited by its cross-sectional study design. These two studies reported protective associations between PA and proliferative DR, but they did not indicate the association between PA and newly developed DR. The present study only included type 2 diabetic patients without DR and revealed the protective associations between PA and the consequent risk of newly developed DR.

Possible mechanisms that link PA to the prevention of the progression and remission of microvascular complications in patients with type 2 diabetes reportedly include the following: reduction of blood pressure, improvements in lipid profile, glycemic control, insulin sensitivity, and endothelial function [34–38]. However, our results show that the association is robust after adjusting for sBP, dBP, BMI, and HbA1c level as time-varying variables, suggesting that PA could lower the risk of developing DR via pathways other than blood pressure, BMI, and glycemic control. In Zucker diabetic rats, chronic running exercise alleviated the early progression of nephropathy with the upregulation of nitric oxide synthases and suppression of glycation [12]. This mechanism could contribute to the reduction of the risk of DR by higher PA in patients with type 2 diabetes.

In this study, 184 patients developed DR (incidence ratio, 54.3/1,000 person-years [95% CI, 47.0–62.7]; Table 3). As previously reported in the Japan Diabetes Complications Study [39], the incidence of diabetic retinopathy in Japanese patients with type 2 diabetes was 38.3/1,000 person-years. The HbA1c levels, age, and duration of diabetes of the patients in this study were 7.8%, 58.2 years old, and 9.8 years, respectively. Although our study participants had better baseline glycemic control, they tended to have a higher age and longer duration of diabetes. These factors could be responsible for the difference in the incidence of DR.

Our results were derived from an observational study, but it is intriguing to consider that PA may play a protective role in the pathogenesis of DR. This hypothesis should be tested in randomized controlled trials designed to examine the effects of altering PA on DR development. Further long-term studies, both clinical and experimental, are needed to establish the clinical utility of PA for the prediction of DR development and to understand the potential pathophysiologic roles of physical inactivity on DR.

Our study had some limitations. First, because this was an epidemiologic study, residual confounders may have existed in the association with DR development and PA that may have acted as bystanders or epiphenomena. Second, the data were derived from the registry of a single diabetes center in Japan, thereby raising concerns regarding generalizations derived from the results, particularly for multiethnic populations. Third, we evaluated only DR development in patients without DR, but did not evaluate changes in the severity of existing DR. Fourth, we were unable to investigate the prevalence of dementia among the participants in our study. Dementia may be related to PA levels or DR. Fifth, the relationship between baseline PA and DR development was estimated in a cohort of patients without DR at baseline. We did not evaluate PA as a time-varying variable because PA was not evaluated every year in this investigation. Moreover, we used subjective estimates to evaluate PA levels instead of assessing PA objectively, for example by using accelerometers. However, the IPAQ is a widely used objective questionnaire. The criterion validity of the short version of the IPAQ had a median rho of about 0.3 [31]. Thus, we believe that the data obtained were trustworthy.

## Conclusion

In conclusion, higher PA was independently associated with a lower incidence of DR in patients with type 2 diabetes. More research needs to be performed to determine if effective strategies to increase PA will reduce the risk of DR in patients with diabetes.
